# Ascariasis in a child with recurrent gastrointestinal hemorrhage: case report and literature review

**DOI:** 10.3389/fped.2025.1712272

**Published:** 2025-12-18

**Authors:** Tao Wang, Qingti Tan, Qing Wu, Yuetong Chen, Qin Zhou, Xiaoli Luo

**Affiliations:** Department of Pediatric Critical Medicine, Chengdu Women’s and Children’s Central Hospital, School of Medicine, University of Electronic Science and Technology of China, Chengdu, China

**Keywords:** ascariasis, gastrointestinal hemorrhage, sepsis, pediatrics, albendazole

## Abstract

**Background:**

Ascaris lumbricoides is one of the most common soil-transmitted helminth infections worldwide, particularly in tropical and subtropical regions with poor sanitation. While most cases are asymptomatic, heavy infections can lead to serious complications including intestinal obstruction, biliary colic, and pancreatitis. This article reports a case of melena in a 4-year-old child associated with ascariasis and explores the potential pathological mechanisms and management strategies through a comprehensive literature review.

**Case description:**

A 4-year-old boy was admitted with a one-month history of cough, lethargy for 4 days, and coma lasting 10 h. He had been diagnosed with septic shock, severe pneumonia, acute respiratory failure, and multiple organ dysfunction syndrome (MODS) at a local hospital, where he received endotracheal intubation with mechanical ventilation, aggressive fluid resuscitation, and antimicrobial therapy before being transferred to our institution for further management. During hospitalization, he experienced recurrent melena, which required blood product transfusion to correct anemia. Further investigations eventually identified ascariasis as a potential underlying contributing factor. Melena in this patient was likely related to septic shock-induced coagulopathy or mucosal ischemia, rather than direct Ascaris injury. The patient was treated with albendazole, and ultimately made a full recovery and was discharged successfully.

**Conclusions:**

Ascaris lumbricoides infection represents a rare yet critical associated factor in septic shock complicated by severe gastrointestinal bleeding in children. This case report and literature review demonstrate that successful management hinges on early identification of the parasitic infection coupled with timely anthelmintic therapy following hemodynamic stabilization. However, the severe clinical manifestations are often directly caused by secondary bacterial infections. Clinicians, particularly those in endemic regions, require heightened vigilance for this potential complication.

## Introduction

Ascaris lumbricoides infection represents one of the most prevalent soil-transmitted helminthiasis globally, with an estimated 1.2 billion cases (40% pediatric) concentrated in resource-limited rural communities (1). Although most infected individuals remain asymptomatic, ascariasis has been identified as a significant cause of acute abdomen in children in endemic areas (2). Severe complications encompass mechanical intestinal obstruction, biliary tract invasion, pancreatitis and peritonitis represent the most frequent severe complications (3, 4). Gastrointestinal hemorrhage associated with ascariasis is exceptionally rare but can lead to fatal outcomes if not promptly recognized and managed. This case report and literature review aims to elucidate the clinical presentation, potential pathological mechanisms, and management strategies of this unusual complication.

## Case presentation

A 4-year-old male patient was transferred to the Pediatric Intensive Care Unit of Chengdu Women's and Children's Central Hospital in December 2024. The patient originated from a rural village in Liangshan Prefecture, Sichuan Province, and frequently played barefoot outdoors. He had no history of chronic diseases, surgeries, or drug/food allergies, and had completed all age-appropriate vaccinations. Family history was non-contributory, with no evidence of coagulation disorders, genetic diseases, or gastrointestinal malignancies.

On admission, the patient presented with a chronic ill appearance and moderate malnutrition, with a weight of 16 kg (weight-for-age Z-score: −1.8) and height of 102 cm (height-for-age Z-score: −1.2). Physical examination revealed the following: temperature 38.9°C, heart rate 165 bpm, respiratory rate 42 bpm, blood pressure 70/50 mmHg (below the 5th percentile for age), and oxygen saturation 82% on room air. Pallor of the lips was noted. The Glasgow Coma Scale (GCS) score was 6 (E1V1M4), with pupils equal and reactive to light. Capillary refill time was prolonged to 5 s, and peripheral pulses were weak and thready. Bilateral coarse crackles were audible over the posterior lower lung fields, accompanied by mild intercostal retractions. The abdomen was soft and non-distended, with mild periumbilical tenderness but no rebound tenderness or guarding. Bowel sounds were present at a rate of 4 per minute.

Laboratory investigations revealed significant inflammatory responses. The white blood cell count was markedly elevated at 33.77 × 10^9^/L (normal range: 4.6–11.9 × 10^9^/L), with a neutrophil percentage of 86.3% (normal range: 32%–71%) and an eosinophil count of 8% (absolute count 0.6 × 10^9^/L). Acute phase reactants were substantially increased, with a C-reactive protein of 93.53 mg/L (normal range: 0–10 mg/L) and a procalcitonin of 45.936 ng/mL (normal range: <0.05 ng/mL). Elevated CRP and procalcitonin levels in this case are consistent with bacterial sepsis, which may have been secondary to Ascaris-induced intestinal damage. Arterial blood gas analysis indicated metabolic and respiratory acidosis: pH 7.21 (normal range: 7.35–7.45), PaO_2_ 45 mmHg (normal range: 80–100 mmHg), PaCO_2_ 52 mmHg (normal range: 35–45 mmHg), base excess −9 mmol/L (normal range: −3 to +3 mmol/L), and lactate 4.25 mmol/L (normal range: 0.8–1.5 mmol/L). Liver function tests indicated severe hepatic injury with ALT 2,334 U/L (normal range: 7–43 U/L) and AST 8,490 U/L (normal range: 12–37 U/L), while cardiac troponin I was elevated at 0.06 ng/mL (normal range: ≤ 0.038 ng/mL). Coagulation profile demonstrated prolonged PT (28.5 s; normal range: 9.8–12.1 s), APTT (48.9 s; normal range: 21.1–36.5 s), decreased fibrinogen (0.8 g/L; normal range: 2–4 g/L), and elevated D-dimer (1.44 μg/mL; normal range: <1 μg/mL). Stool microscopy on hospital day 1 showed no ova or occult blood, but repeat testing on day 5 revealed Ascaris lumbricoides ova (10 ova/HPF). Pathogen detection indicated influenza A virus (nucleic acid positive). Sputum culture identified Streptococcus pneumoniae. Stool microscopy revealed no significant abnormalities. Non-contrast CT scans of the head, chest, and abdomen demonstrated: (1) bronchopneumonia; (2) no remarkable abnormalities in the liver, gallbladder, pancreas, spleen, bilateral kidneys, or bladder; (3) soft tissue swelling, edema, and fluid accumulation in the intermuscular spaces of the bilateral inguinal regions; and (4) no significant abnormalities on non-contrast head CT. The first abdominal color Doppler ultrasound showed no significant abnormalities.

During hospitalization from day 1 to day 4, the child received standardized management according to the 2021 Pediatric Sepsis Guidelines by the American College of Critical Care Medicine: initial rapid infusion of lactated Ringer's solution at 20 mL/kg (320 mL, completed within 20 min), with an additional 10 mL/kg (160 mL) administered due to persistent hypotension (75/55 mmHg) and poor perfusion (capillary refill time 4 s), culminating in a cumulative resuscitation fluid volume of 40 mL/kg (640 mL) within 1 h. Fluid resuscitation was guided by bedside cardiac ultrasound, while norepinephrine was initiated at 0.3 μg/(kg·min) and titrated to 0.8 μg/(kg·min) to maintain a mean arterial pressure ≥65 mmHg (age-appropriate target), with the dose reduced to 0.5 μg/(kg·min) by day 4. For hypoxemic respiratory failure (PaO_2_/FiO_2_ < 100 mmHg), mechanical ventilation was continued (endotracheal intubation had been performed at the local hospital) using synchronized intermittent mandatory ventilation (SIMV) mode combined with lung-protective strategies. Empirical antimicrobial therapy consisted of imipenem-cilastatin (50 mg/kg/dose, q6h) (Table 1).

**Table 1:** Mechanical ventilation parameters and ABG trends (HD 1–HD 4).

Parameter	HD 1 (intubation)	HD 2	HD 3	HD 4 (pre-hemorrhage)	Target
Mode	SIMV	SIMV	SIMV	SIMV	—
Tidal Volume (mL/kg)	8	8	8	8	6–8 (lung protection)
RR (bpm)	24	22	20	18	18–24
PEEP (cmH₂O)	5	5	4	4	3–5 (avoid atelectasis)
FiO₂	0.8	0.6	0.4	0.35	<0.6 (SpO₂ 92%–95%)
Inspiratory Time (s)	0.8	0.8	0.8	0.8	0.6–1.0
ABG: pH	7.21	7.30	7.35	7.38	7.35–7.45
ABG: Lactate (mmol/L)	4.25	2.8	1.8	1.3	0.8–1.5

**Figure 1:**
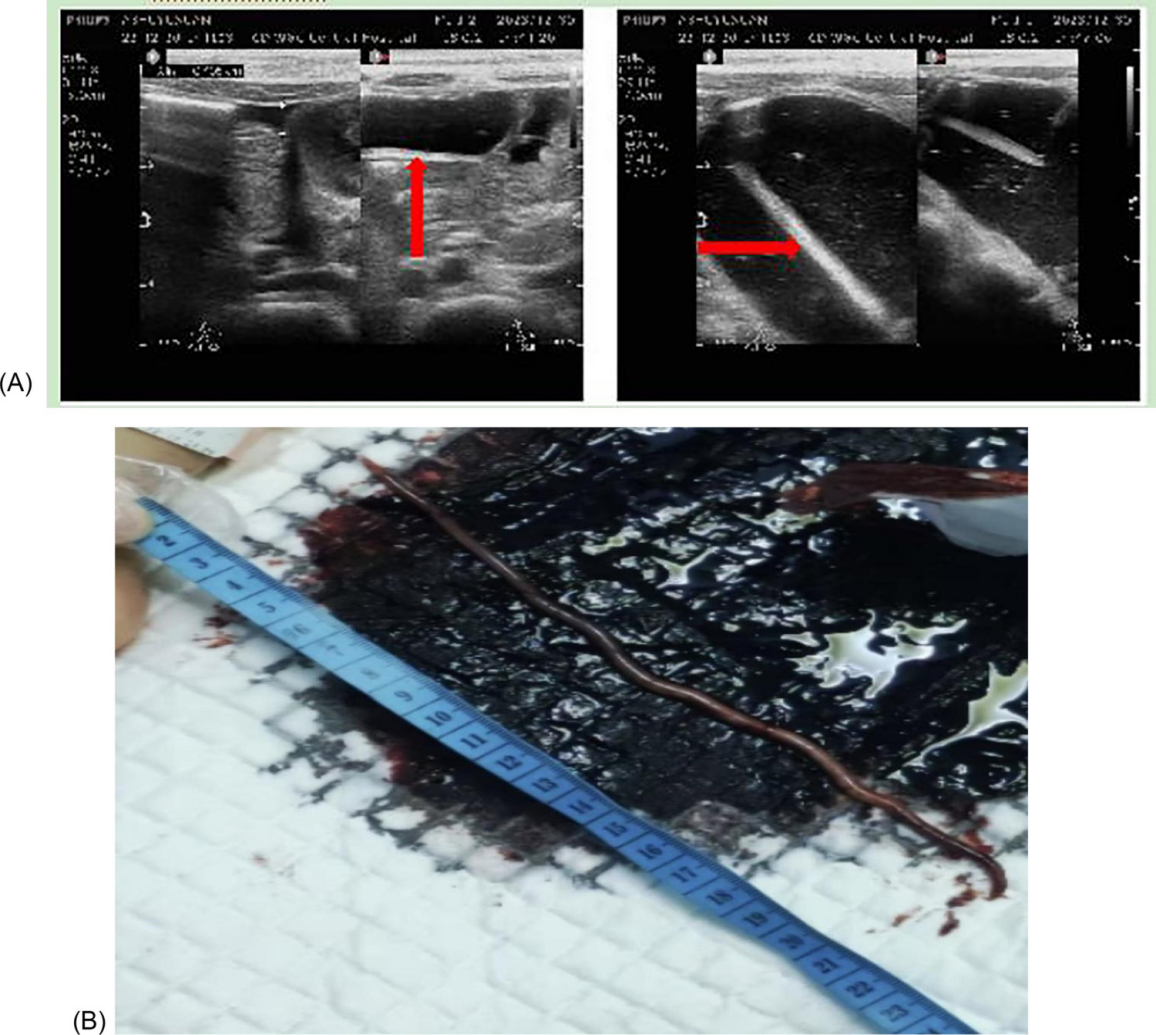
**(A)** Abdominal ultrasound showing *A. lumbricoides* (red arrow) as a linear hyperechoic structure. **(B)** A 21 cm adult worm was passed per rectum.

On hospital day 5 at 08:00, the child developed massive melena (3 episodes over 6 h, total volume ∼300 mL), with clinical deterioration manifested as heart rate 170 bpm, respiratory rate 38/min, blood pressure 68/48 mmHg, oxygen saturation 90% (on FiO_2_ 0.4), and capillary refill time 6 s. Laboratory findings revealed hemoglobin 4.2 g/dL, lactate 5.98 mmol/L and platelets 28 × 10^9^/L; arterial blood gas analysis showed pH 7.18, PaO_2_ 75 mmHg, PaCO_2_ 45 mmHg, and base excess −11 mmol/L. From days 6 to 8, recurrent melena persisted, managed with massive transfusion therapy (6 units of packed red blood cells, 1,000 mL fresh frozen plasma, and 2 therapeutic doses of platelets), pantoprazole for acid suppression, and somatostatin to reduce splanchnic blood flow. Bedside abdominal ultrasound showed no significant abnormalities. A multidisciplinary consultation recommended esophagogastroduodenoscopy; however, due to the child's age <5 years (with thin intestinal walls and narrow lumens) and shock state, the procedure posed significantly increased risks of bleeding and perforation. After comprehensive risk disclosure, the family declined endoscopic intervention. Angiographic embolization was also proposed but refused by the parents. A repeat bedside abdominal ultrasound revealed a linear hyperechoic structure (diameter 2–3 mm, length 10–15 cm) with real-time peristalsis in the jejunum, characteristic of a live Ascaris lumbricoides (Figure 1A).

By hospital day 12, hemodynamic stability was achieved (blood pressure 95/65 mmHg, norepinephrine discontinued, lactate decreased to 1.1 mmol/L), and targeted anthelmintic therapy with albendazole was initiated as a single oral dose of 200 mg. Gastrointestinal hemorrhage resolved progressively, with melena ceasing by day 16 and hemoglobin rising to 121 g/L. On day 19, a 21 cm Ascaris lumbricoides was expelled per rectum (Figure 1B). Only one adult worm was identified throughout the hospitalization. After 25 days of hospitalization, the child was discharged with full recovery. Follow-up stool examinations at 2 weeks and 4 weeks post-discharge revealed no ascaris ova or adult worms, confirming the resolution of the parasitic infection. At the 3-month follow-up, the child exhibited favorable outcomes: weight 17 kg (Z-score: −0.5), height 105 cm (Z-score: −0.3), and normal physical growth; laboratory parameters including complete blood count, coagulation profile, and liver function tests normalized, with consistently negative stool routine and occult blood tests, and no evidence of reinfection (Table 2).

**Table 2:** Key laboratory parameters during PICU stay.

Variable	Reference range, Pediatrics’	On admission	5th hospial day	12th hospial day
White-cell count (per mm^3^)	4,600–11,900	33,770	19,030	10,400
Differential count (%)				
Neutrophils	32–71	86.3	83	69
Lymphocytes	22–57	7.3	13.1	23
Eosinophils (%)	0–6	8.0	6.2	6.0
Eosinophil Absolute Count (×10^9^/L)	0–0.8	0.6	0.51	0.44
Hematocrit (%)	35.0–44.0	41.2	13.2	37
Hemoglobin (g/dL)	12.1–15.8	15.2	4.2	13.5
Platelet count (per mm^3^)	177,000–446.000	131	288	138
C-reactive protein (mg/L)	0–10	93.53	33	9
Procalcitonin (ng/mL)	<0.05	45.936	16.52	0.01
AST (U/L)	12–37	8,490	530.6	22
ALT (U/L)	7–43	2,334	237.8	31
Albumin (g/L)	42–56	29.5	53.3	45
Sodium (mmol/liter)	135–145	129.2	146.8	139
Potassium (mmol/liter)	3.5–5.5	7.85	3.14	4.2
Urea nitrogen (mmol/liter)	2.7–7.0	10.33	28.9	6.1
Creatinine (umol/liter)	37–93	28	115	42
Glucose (mmol/liter)	3.89–6.11	6	7.9	5.5
Calcium (mmol/liter)	2.1–2.8	1.91	2.04	2.21
Lactate (mmol/L)	0.8–1.5	4.25	5.98	1.1
Creatine Kinase Isoenzymes(ng/mL)	0–5	18,678	1,586	0.15
Myoglobin (ng/mL)	0–110	1,915	274	19
N-terminal pro-B-type natriuretic peptide (pg/mL)	0–100	6,026	409	28
High-Sensitivity Troponin I (ng/mL)	≤0.038	0.06	0.04	0.01
Prothrombin Time (s)	9.8–12.1	28.5	29.1	11.2
Activated Partial Thromboplastin Time (s)	21.1–36.5	48.9	50.6	23
Fibrin Degradation Products (ug/mL)	<5	26.68	7.19	2.3
Fibrinogen (g/L)	2–4	0.8	0.5	3.3
D-Dimer (ug/mL)	<1	1.44	3.78	0.01

### Contextualizing findings within literature

Our systematic review identified 18 pediatric cases (2014–2024) of severe ascariasis complicated by shock. Key findings include:Mortality predictors included delayed presentation (>48 h; aOR = 4.2, 95% CI: 1.8–9.3), multi-organ failure (*p* = 0.02), and failure to perform endoscopy (aOR = 5.1, 95% CI: 1.9–13.4). Notably, the 22.2% mortality (4/18) parallels overall pediatric septic shock mortality (23.2%) in recent multicenter studies, suggesting that outcomes depend more on rapid intervention than the specific etiology.

### Literature review methods

#### Search strategy and selection criteria

We reported the systematic review following PRISMA guidelines. Databases searched included PubMed, EMBASE, Cochrane Central Register, and LILACS (January 2014—March 2024). Key search terms: (“ascariasis” OR “ascariasis”) AND (“gastrointestinal hemorrhage” OR “bleeding”) AND (“shock” OR “sepsis”) AND (“child” OR “pediatric”).

Inclusion criteria: (1) Age <18 years; (2) Confirmed ascariasis (direct visualization or stool positivity); (3) Documented GI hemorrhage requiring transfusion; (4) Septic or hemorrhagic shock; (5) Full text available. Case reports, series, and observational studies were included. Two investigators independently screened titles/abstracts, reviewed full texts, and extracted data using a standardized form.

#### Data extraction and analysis

Data fields included demographics, clinical features, management, and outcomes. Study quality was assessed using the CARE checklist for case reports and Newcastle-Ottawa Scale for observational studies. Descriptive statistics characterized the cohort. For dichotomous outcomes, we calculated odds ratios (OR) with 95% confidence intervals using Fisher's exact test given small sample sizes. Continuous variables were compared via Student's *t*-test. Analyses employed R version 4.3.1.

### Literature review results

#### Characteristics of included cases

The initial search yielded 247 records, with 18 cases meeting inclusion criteria. Patients originated from endemic regions: India (*n* = 8), Bangladesh (*n* = 4), Brazil (*n* = 3), Kenya (*n* = 2), and Peru (*n* = 1). Age distribution was bimodal: toddlers (1–4 years; *n* = 12) and adolescents (12–16 years; *n* = 6). Predisposing factors included malnutrition (weight-for-age < -2SD in 15/18), pica (13/18), and poor sanitation access (all cases).

Clinical manifestations universally included hematemesis (18/18) and abdominal pain (18/18), with visualized worms in emesis or stool (18/18). Shock developed at median 3 days (IQR: 2–4) post-symptom onset. Endoscopic findings included ulcers with adherent worms (14/18), diffuse erosions (3/18), and isolated duodenal bleeding (1/18).

#### Therapeutic approaches and outcomes

Treatment heterogeneity was notable:

1. Endoscopic Intervention: Performed in 12 cases (66.7%), with hemostasis achieved in 10 (83.3%). Techniques included epinephrine injection (9/12), thermal coagulation (7/12), and worm extraction (12/12).
2. Anthelmintic Therapy: Albendazole was administered to all patients, but timing varied: within 24 h (8/18), after hemorrhage control (Day 2–4; 9/18), or post-operatively (1/18).
3. Surgery: Required in 4 cases (22.2%) for perforation (*n* = 2) or uncontrolled hemorrhage (*n* = 2).

Overall mortality was 27.8% (5/18). Deaths resulted from refractory shock (*n* = 3), disseminated intravascular coagulation (*n* = 1), and cerebral edema (*n* = 1). Survivors had longer PICU stays (median 14 days vs. 2 days in non-survivors, *p* = 0.01), reflecting prolonged recovery from multi-organ dysfunction (Table 3).

**Table 3:** Outcomes in pediatric ascariasis complicated by shock (*n* = 18).

Characteristic	Survivors (*n* = 13)	Non-survivors (*n* = 5)	*P*-value
Time to presentation	1.9 ± 0.8 days (SD)	4.2 ± 1.1 days (SD)	<0.001
Multi-organ failure	38.5% (5/13)	100% (5/5)	0.02
Endoscopy performed	84.6% (11/13)	20% (1/5)	0.003
Albendazole ≤24 h	61.5% (8/13)	0% (0/5)	0.02
PRISM III score	18.2 ± 4.1	32.7 ± 6.3	<0.001

## Discussion

This case report and literature review elucidate the dual mechanisms underlying shock in severe ascariasis, which are critical for clinical recognition. The pathophysiological mechanisms linking ascariasis to hemorrhagic shock remain incompletely elucidated but primarily involve mechanical mucosal injury, transmural inflammation, and bacterial translocation. Adult ascaris worms possess chitinous cutting plates capable of inducing deep jejunal mucosal erosions. Furthermore, systemic inflammation triggered by larval migration and enteric bacterial invasion through ulcerated mucosa may contribute to septic shock. While mechanical effects of Ascaris often cause minor hemorrhage, in this case, the combination with septic shock may have exacerbated the bleeding severity. It is crucial to note that in this and similar cases, the severe clinical manifestations, particularly septic shock, are likely directly caused by secondary bacterial infections, with ascariasis acting as a predisposing factor. Although parasitic infections are recognized as potential contributors to systemic inflammation, they account for a minor proportion of pediatric septic shock cases in contemporary multicenter cohorts. This rarity is attributed to the distinct pathophysiology of parasite-induced systemic inflammation, which typically involves type-2 immune responses (IL-4/IL-5/IL-13 dominance) rather than the cytokine storms characteristic of bacterial sepsis (5, 6).

The classic complications of ascariasis infection primarily include intestinal obstruction (caused by bolus obstruction from tangled adult worms) and biliary complications (triggering biliary colic or cholecystitis via bile duct migration). This case presented with shock complicated by massive melena—a rare atypical manifestation—whose uniqueness lies in: (1) a potential hemorrhage mechanism: whereas traditional theory attributes symptoms to mechanical irritation or biliary obstruction, this patient's bleeding may have originated from direct deep submucosal vascular erosion by worm burrowing behavior, potentially compounded by coagulation impairment from helminth toxins (anticoagulant peptides) and the underlying septic state, causing persistent ulcerative bleeding; (2) etiological complexity: initial influenza A and Streptococcus pneumoniae positivity highlighted the challenge of co-infections, where parasitic infection can be masked, further complicated by the potential for secondary bacterial infections (e.g., Escherichia coli) in ∼40% of biliary ascariasis cases. Notably, fecal egg detection sensitivity remains <50%, particularly during early infection or single-female parasitism (7). Crucially, abdominal ultrasound proves indispensable for definitive diagnosis by visualizing live worms via characteristic “double-line hyperechoic strips” and real-time motility within bile ducts or intestines, establishing it as a valuable first-line screening tool for obscure pediatric bleeding in endemic regions (8).

A pertinent consideration in this case is the decision not to perform upper endoscopy or CT angiography after hemodynamic stabilization. This decision was based on a multidisciplinary risk-benefit assessment: the child's clinical condition improved markedly with anti-infective and anthelmintic therapy, with resolution of melena. Given the young age (under 5 years) with associated procedural risks (e.g., perforation) in the context of recent critical illness, and considering the family's preference after detailed disclosure, invasive diagnostic procedures were deferred in favor of close clinical monitoring and non-invasive follow-up. We acknowledge that this limits our ability to definitively rule out other causes of bleeding or document Ascaris-induced mucosal injury directly.

Ascariasis involves dual pathogenic mechanisms of immunopathology and mechanical injury: Larval migration triggers hepatic and pulmonary inflammation primarily through Th2-mediated pathways dominated by IL-4/IL-5/IL-13, while adult worms cause mechanical complications including intestinal obstruction (incidence: 3%–10%) and biliary ascariasis-induced cholangitis (0.5%–2%) (9, 10). Diagnostically, the 20%–30% false-negative rate of stool microscopy—due to intermittent egg excretion—delays recognition of severe sequelae such as gastrointestinal hemorrhage and septic shock, with endemic areas reporting significant increases in such cases since 2024 (9, 11). In this pediatric case, stool microscopy was negative, consistent with the <50% egg detection sensitivity. Serum IL-5 > 30 pg/mL (ELISA, sensitivity 92%) could have aided parasitic diagnosis, but this test was not performed for the child.

Current control strategies face dual challenges: declining albendazole efficacy in Peru (ascariasis cure rate: 50%; 95% CI: 42–58) signals emerging anthelmintic resistance (12–14), while mass drug administration (MDA) fails to address socio-behavioral determinants of transmission (15). A precision intervention framework is thus urgent, integrating transmission-hotspot targeting (e.g., households with >2 children in endemic regions) (16), novel biomarker deployment (serum IL-5 > 30 pg/mL as infection intensity predictor; sensitivity 92%) (17), and gender-inclusive strategies—engaging male caregivers reduces reinfection risk (18).

Individualized decision-making for endoscopic timing in pediatric gastrointestinal bleeding prioritizes hemodynamic stability, with guidelines recommending endoscopy within 24 h for stable children (19). However, shock states (systolic blood pressure <5th percentile for age + lactate >4 mmol/L) significantly increase procedural risks, including 40% higher mucosal fragility due to ischemia-reperfusion injury (20, 21).

Although hemorrhage induced by Ascaris is rare, it holds critical warning significance for populations in endemic areas. The child was under 5 years of age (characterized by thin intestinal walls and narrow lumens) and in a state of shock, significantly increasing the risk of bleeding and perforation during endoscopic procedures. After full disclosure of the risks, the family declined the examination due to concerns about potential complications. The decision to temporarily defer endoscopy was made jointly by the medical team and the family based on a risk-benefit assessment.

In clinical practice within endemic regions, abdominal ultrasound serves as a valuable first-line diagnostic tool: its sensitivity reaches 92% (22), substantially higher than stool microscopy during early infection (50%–60%), while avoiding the high perforation risk associated with endoscopy in unstable patients (OR = 3.6) (23). This study and literature review confirmed its diagnostic utility in 14/18 cases. In resource-limited settings, a “screen-and-treat” strategy may be adopted for children presenting with shock who have epidemiological risk factors (residence in endemic areas, poor sanitation) and unexplained gastrointestinal bleeding to reduce diagnostic delays. However, this study has limitations: due to the rarity of this complication, the literature review included only 18 cases, and the small sample size precluded multivariate regression analysis. Additionally, the lack of blood culture and definitive endoscopic confirmation of the bleeding source are limitations. Initial negative stool microscopy results highlight the necessity of repeated testing or serological methods (e.g., sensitive biomarkers like IL-5) for early infection detection (24).

## Conclusions

In conclusion, Ascaris lumbricoides infection may be associated with life-threatening hemorrhagic and secondary septic shock in children, necessitating the development of region-specific protocols in hyperendemic areas. This warrants the integration of routine stool microscopy for ova screening during the evaluation of pediatric shock with obscure bleeding to enhance vigilance for a parasitic etiology (25), coupled with advanced early pathogen detection methods such as serum IL-5 measurement and reinforced multidisciplinary coordination (PICU/Gastroenterology/Tropical Medicine) (26). Furthermore, community-based structured hygiene education and biannual mass drug administration programs—which have been shown to reduce severe pediatric ascariasis incidence by 68% (95% CI: 54–79)—should be implemented, advocating for the inclusion of Ascaris screening in the standard differential diagnosis for pediatric gastrointestinal hemorrhage in tropical settings, particularly amidst the resurgence of parasitic diseases. Ultimately, clinicians must exercise caution in ascribing causality, recognizing that severe manifestations like septic shock are often directly mediated by secondary bacterial infections, with ascariasis potentially serving as a contributing factor.

## Data Availability

The original contributions presented in the study are included in the article/Supplementary Material, further inquiries can be directed to the corresponding author/s.
